# A synopsis of *Harperocallis* (Tofieldiaceae, Alismatales) with ten new combinations

**DOI:** 10.3897/phytokeys.21.4859

**Published:** 2013-05-09

**Authors:** Lisa M. Campbell, Laurence J. Dorr

**Affiliations:** 1 The New York Botanical Garden, Bronx, New York 10458, USA; 2 Department of Botany, National Museum of Natural History, MRC-166, Smithsonian Institution, P.O. Box 37012, Washington, D.C. 20013–7012, USA

**Keywords:** Andes, Coastal Plain, Florida, Guayana region, *Isidrogalvia*, monocot, nomenclature, *Tofieldia*

## Abstract

Ten new combinations from *Asagraea*, *Isidrogaliva*, and *Tofieldia* are proposed in the previously monospecific genus *Harperocallis* (Tofieldiaceae, Alismatales). As circumscribed here, the genus is restricted to the Americas. The majority of species occur in the Andes or the Guayana region of northern South America; more than half have restricted distributions, and *Harperocallis flava* is narrowly endemic in the Coastal Plain of the southeastern United States. A key to species, synonymies, distributions, representative specimens, and salient notes are presented. Populations of the species are mapped and *Harperocallis robustior* is illustrated. A neotype is selected for *Tofieldia frigida*, here considered a synonym of *Harperocallis falcata*. Several recent records of *Harperocallis longiflora*, previously known only from the type collected in 1902, are reported.

## Abstract (es)

Se proponen diez nuevas combinaciones de *Asagraea*, *Isidrogaliva*, y *Tofieldia* en *Harperocallis* (Tofieldiaceae, Alismatales), un género previamente monoespecífico. Como se circunscribe aquí, el género se restringe a las Américas. La mayoría de las especies se encuentran en los Andes o en la región de Guayana en el norte de América del Sur. Más de la mitad tienen distribuciones restringidas, y *Harperocallis flava* es estrictamente endémica en la llanura costera del sudeste de los Estados Unidos. Se presenta una clave para distinguir las especies, sinonimias, distribuciones, muestras representativas y notas salientes. Se mapearon poblaciones de las especies y se ilustra *Harperocallis robustior*. Se seleccionó un neotipo para *Tofieldia falcata*, considerado aquí como un sinónimo de *Harperocallis falcata*. Se reportan algunos registros adicionales de *Harperocallis longiflora*, especie anteriormente conocida sólo con el tipo colectado en 1902.

## Introduction

When Ruiz and Pavon (1802) established the genus *Isidrogalvia* Ruiz & Pav. they described and illustrated a single species, *Isidrogalvia falcata* Ruiz & Pav. This species was collected in Peru, but it is clear from their protologue that they did not intend to recognize a genus restricted to South America as they stated that the European species *Anthericum calyculatum* L. should be referred to their new genus (“Ad hoc Genus referendum est *Anthericum calyculatum* Linn.”). This European species is the type of the generic name *Tofieldia* Huds., which when published by Hudson (1787) included only one named species, *Tofieldia palustris* Huds., a name superfluous for, and homotypic with, *Anthericum calyculatum* (McNeill et al. 2012; Arts 7.5 & 52). Thus, Ruiz and Pavon (1802) inadvertently created a generic synonym of *Tofieldia*.

The nomenclatural implications of this synonymy were overlooked when the South American taxa were revised by Cruden (1991) and when additional South American taxa were transferred to or described in *Isidrogalvia* (Cruden and Dorr 1992, Remizowa 2007, Campbell 2010). Concurrently, recognition of a distinct South American genus was reinforced as palynological (Mosyakin et al. 2009, Campbell 2010), morphological (Remizowa et al. 2010, 2011), and molecular data (Azuma and Tobe 2011) revealed that these South American taxa were distinct from *Tofieldia*. Phylogenetic analyses of molecular (Azuma and Tobe 2011) and morphological data (Remizowa et al. 2011) resolved the monospecific North American *Harperocallis* McDaniel (1968) as sister to the South American taxa (Azuma and Tobe 2011), or as derived with that lineage, and *Harperocallis flava* McDaniel was transferred to *Isidrogalvia* (Remizowa et al. 2011). Long known from only three populations, recent field surveys revealed additional populations (Leonard and Baker 1983, Walker and Silletti 2005, Keppner and Anderson 2008) of this endangered species (US Fish and Wildlife Service 1991, see also Pitts-Singer et al. 2002). *Harperocallis flava*, nonetheless, remains narrowly endemic and has low infraspecific genetic diversity (Godt et al. 1997).

Due to the nomenclatural consequences of Ruiz and Pavon’s synonymy (1802), Sokoloff et al. (2011) proposed conservation of the genus *Isidrogalvia* with a conserved type, *Isidrogalvia falcata*, a reasonable solution given that all recent literature treating the South American taxa (see Sokoloff et al. 2011) had employed the generic name *Isidrogalvia*. This proposal, however, was rejected (Applequist 2012) as the Nomenclature Committee for Vascular Plants indicated that they preferred to follow the rule of priority (McNeill et al. 2012; Art. 11.4). Consequently, we propose the following ten transfers of names first published in *Asagraea* Lindl., *Isidrogalvia*, or *Tofieldia* to *Harperocallis*.

## Methods

Herbarium specimens or their images (indicated by an identifier in brackets) were examined from the following herbaria (herbarium abbreviations follow Index Herbariorum, http://sweetgum.nybg.org/ih): A, B-W (Röpert 2000), BC (Courtesy of JSTOR 2012), BM (Courtesy of JSTOR 2012), BRIT (BRIT Virtual Herbarium), F (The Field Museum 2013), FLAS (Florida Museum of Natural History 2013), FSU (Mast et al. 2004), FTG, GH, K (Courtesy of JSTOR 2012), MA (Courtesy of JSTOR 2012), MO, NY, P (Muséum National d’Histoire Naturelle 2012), PH (Courtesy of JSTOR 2012), PORT, US, and VEN. Measurements were obtained visually and augmented from literature (*Asagraea* and *Harperocallis flava*).

## Nomenclatural synopsis

### Harperocallis

McDaniel, J. Arnold Arbor. 49 (1): 36. 1968.

http://species-id.net/wiki/Harperocallis

Figs 1 –3

#### Type.

*Harperocallis flava* McDaniel.

#### Key to the Species of *Harperocallis*

(modified from: Cruden 1991, and Campbell 2010)

**Table:** 

1	Capsules ribbed or unribbed, well-developed lateral veins never extending to the style base	6
–	Capsules 9-ribbed, well-developed lateral veins extending to the style base	2
2	Inflorescence compound, the branches subtended by chlorophyllous cataphylls	*Harperocallis paniculata*
–	Inflorescence a simple raceme; peduncular bracts scarious	3
3	Flowers pendant	*Harperocallis penduliflora*
–	Flowers erect	4
4	Leaves 2.0–4.0 mm wide, usually glabrous, occasionally ciliate at the sheath apex; peduncles < 1.5 mm wide; calycular bracts longer than wide; tepals 6.0–9.0 **×** 1.5–2.0 mm; anthers 0.9–1.1 mm long	*Harperocallis duidae*
–	Leaves 3.0–7.0 mm wide, margin ciliolate to hirsutulous; peduncles > 1.5 mm wide; calycular bracts usually wider than long, sometimes equal; tepals 11–14 **×** 2–4 mm; anthers > 1.1 mm long	5
5	Leaves 3.0–6.5 mm wide; peduncular bracts 3–8 (–10); inflorescence ca. 9–30-flowered; tepals enclosing the rest of the flower; anthers 1.2–1.8 mm long	*Harperocallis schomburgkiana*
–	Leaves 6.0–7.0 mm wide; peduncular bracts 3 or 4; inflorescence ca. 25–40-flowered; tepals open; anthers 2.0–2.5 mm long	*Harperocallis neblinae*
6	Capsule incompletely 9-ribbed, well-developed lateral veins never extending to the style base	7
–	Capsule 3-ribbed or unribbed	8
7	Inflorescence with eglandular hairs; peduncular bracts (3–) 4–11 (–15); flowers (4–) 8–28; styles recurved, apices oblique; stigmas facing outward; capsule etuberculate	*Harperocallis sessiliflora*
–	Inflorescence glabrous; peduncular bracts 7–17; flowers 23–35; styles straight; stigmas capitate; capsule tuberculate	*Harperocallis robustior*
8	Leaves glabrous, veins not prominent; inflorescence 1-flowered; flower erect; ovary densely tuberculate; capsule unribbed	*Harperocallis flava*
–	Leaf margin tomentose, ciliolate or glabrous, veins prominent; inflorescence > 1-flowered, or if 1-flowered, the flower pendant; ovary not tuberculate; capsule 3-ribbed	9
9	Peduncular bract solitary, chlorophyllous; inflorescence 1–4-flowered; flowers pendant	*Harperocallis sipapoensis*
–	Peduncular bracts (1) 2–8, scarious; inflorescence > 10-flowered; flowers erect	10
10	Calycular bracts usually longer than wide (1.8–3.0 **×** 1.7–2.5 mm); styles 0.3–0.8 (–0.9) mm long, straight; stigmas capitate	*Harperocallis falcata*
–	Calycular bracts usually wider than long (1.5–2 **×** 1.8–2.3 mm); styles 0.9–1.1 mm long, recurved, apices oblique; stigmas facing outward	*Harperocallis longiflora*

### Harperocallis duidae

(Steyerm.) L.M. Campb. & Dorr comb. nov.

urn:lsid:ipni.org:names:77128356-1

http://species-id.net/wiki/Harperocallis_duidae

Tofieldia duidae Steyerm., Fieldiana, Bot. 28 (1): 156. 1951. Type: Venezuela. Amazonas:stream bank above Vegas Falls, Brocchinia Hills, summit of Cerro Duida, 1700–1980 m, 1 Sep 1944, *J.A. Steyermark 58176* (holotype: F [F0046065F!]; isotypes: MO!, NY!, US!, VEN).
Isidrogalvia duidae (Steyerm.) Cruden, Syst. Bot. 16 (2): 278. 1991. Type: Based on *Tofieldia duidae* Steyerm.

#### Type.

Based on *Tofieldia duidae* Steyerm.

#### Distribution

**Figure 1.**
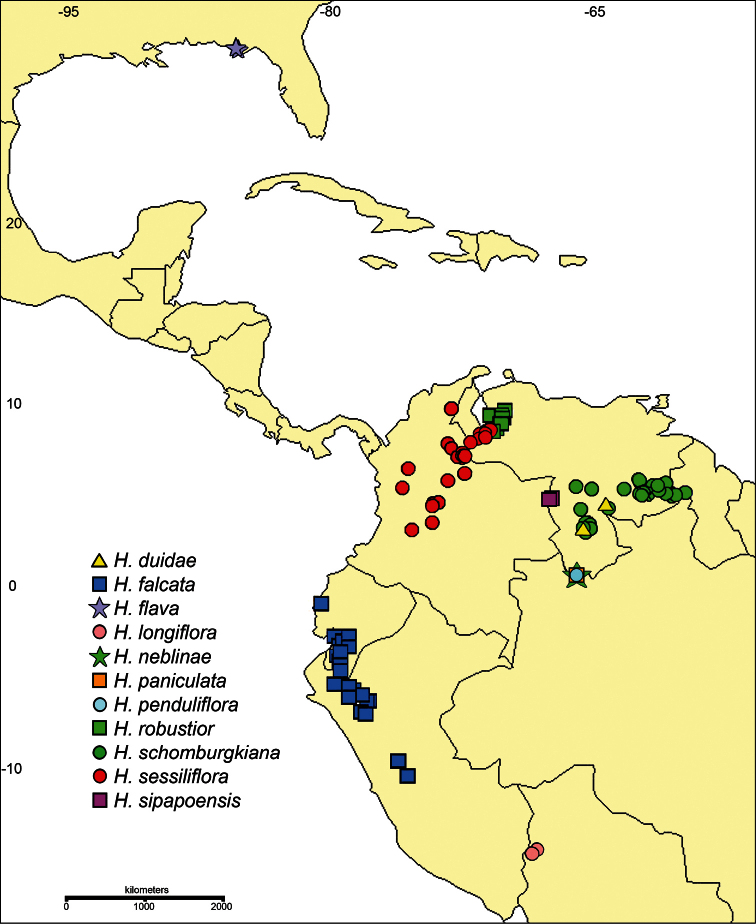
Distribution of *Harperocallis*.

(Fig. 1). Endemic to the Guayana Highlands of Venezuela (Amazonas and Bolívar states) where it is known only from two tepuis (Cerros Duida and Jáua); 1000–2100 m.

#### Representative specimens.

**VENEZUELA**. **Amazonas:** Mpio. Atabapo, Parque Nacional Duida-Marahuaka, Macizo del Duida, 2100 m, Apr 1991, *A. Fernández et al. 8094* (US); Cerro Duida, río Cunucunuma, 1000–1100 m, 18 Nov 1950, *B. Maguire et al. 29515* (NY); along upper Caño Culebra, 1500–1600 m, 21 Nov 1950, *B. Maguire et al. 29616* (NY, US). **Bolívar:** Meseta de Jáua, Cerro Jáua, cumbre de la porción Central-Occidental de la Meseta, 1922–2100 m, 22–27 Mar 1967, *J.A. Steyermark 97899* (NY).

### Harperocallis falcata

(Ruiz & Pav.) L.M. Campb. & Dorr comb. nov.

urn:lsid:ipni.org:names:77128358-1

http://species-id.net/wiki/Harperocallis_falcata

Isidrogalvia falcata Ruiz & Pav., Fl. Peruv. 3: 69, t. 302b. 1802. Type: Peru. de Pillao [sic], *H. Ruiz & J.A. Pavon s.n*. (lectotype: MA [MA810531!], F-negative no. 29435 (F!, MO!), selected by Cruden, 1991; possible isolectotypes: BC [BC872749!], BM [BM000938089!], MA [MA810498!, MA [MA810530!]).
Tofieldia falcata (Ruiz & Pav.) Pers., Syn. Pl. 1: 399. 1805. Type: Based on *Isidrogalvia falcata* Ruiz & Pav.
Tofieldia falcata (Ruiz & Pav.) Willd., Ges. Naturf. Freunde Berlin Mag. Neuesten Entdeck. Gesammten Naturk. 2: 29. 1808, comb. illeg. Type: Based on *Isidrogalvia falcata* Ruiz & Pav.
Narthecium falcatum (Ruiz & Pav.) Poir., Encycl., Suppl. 4: 61. 1816. Type: Based on *Isidrogalvia falcata* Ruiz & Pav.
Asphodeleris falcata (Ruiz & Pav.) Kuntze, Revis. Gen. Pl. 2: 706. 1891. Type: Based on *Isidrogalvia falcata* Ruiz & Pav.
Tofieldia flexuosa Willd., Ges. Naturf. Freunde Berlin Mag. Neuesten Entdeck. Gesammten Naturk. 2: 28. 1808. Type: Peru [Ecuador?]. *F.W.H.A. von Humboldt & A.J.A. Bonpland 3383* (holotype: B-W [B-W-07103-01 0!]; isotypes: GH-fragm., P [P02137266!]).
Tofieldia frigida Kunth in H.B.K., Nov. Gen. Sp. [quarto ed.] 1: 267. 1815 [1816]; Ibid. [folio ed.] 1: 213. 1815 [1816]. Type: Peru [Ecuador?]. *F.W.H.A. von Humboldt & A.J.A. Bonpland 3383* (neotype, here designated: B-W [B-W-07103-01 0!]; isoneotypes: GH-fragm., P [P02137266!]).

#### Type.

Based on *Isidrogalvia falcata* Ruiz & Pav.

#### Distribution

(Fig. 1). The Andes of Ecuador (Azuay, Cañar, Loja, Morona-Santiago, and Zamora-Chinchipe provinces) and Peru (Amazonas, Cajamarca, Cusco, Huánuco, Junín, and Pasco regions); 2300–3860 m.

#### Representative specimens.

**ECUADOR. Azuay:** Cordillera Oriental, alrededores del Páramo de Patococha entre Gualaceo y Limon, 3400–3450 m, 6–7 Aug 1959, *H.G. Barclay & P. Juajibioy 8632* (MO, NY); Km 85 on Pan American Highway N of Loja, 2850–2950 m, 3 May 1973, *L. Holm-Nielsen et al. 4815* (MO, NY); Hac. Horta-Naque, 3100 m, 11 Jun 1946, *H.N. Moldenke 869* (NY). **Cañar:** Cerro Yanguán NE of Pindilig, 3100 m, 13 Dec 1980, *L.B. Holm-Nielsen et al. 29300* (MO, NY). **Loja:** Loma de Oro at Panamerican Highway, 3300 m, 2 Jan 1981, *H. Balslev 1382* (MO, NY, US); Yangana–Zumba road Km 15–20, N slopes of Cordillera de Sabanilla, 2550 m, 31 Dec 1980, *H. Balslev 1298* (NY). **Loja/Zamora-Chinchipe:** Parque Nacional Podocarpus, crest of the Cordillera de los Andes E and SE of Nudo de Cajanuma, s.d., *B. Øllgaard 90768* (NY). **Morona/Santiago:** Gualaceo–Sigsig–Gualaquiza road, SSE of Sigsig, 03°11'S, 78°40'W, ca. 2900–3090 m, 3 Dec 1990, *J.L. Luteyn et al. 14287* (NY). **PERU. Amazonas:** Prov. Chachapoyas: Cerca a Calla-Calla, siguiendo la ruta a Leymebamba–Balsas, 3860 m, 18 Dec 1992, *I. Sanchez Vega & J. Tanta 6481* (US); Cerros [de] Calla Calla, east side, 19 km above Leimebamba [sic] on the road to Balsas, 3100 m, 4 Jun 1964, *P.C. Hutchinson & J.K. Wright 5510* (NY, US). **Cajamarca:** Prov. Cutervo: 2350 m, 22 Jun 1992, *I. Sanchez Vega & A. Miranda 6265* (NY).

#### Notes.

When compared to its present on-line image (http://plants.jstor.org/specimen/ma810531), a photograph (F-negative no. 29435) of the lectotype of *Isidrogalvia falcata* taken by J. Francis Macbride of the Field Museum before World War II shows that the original label (“*Isidrogalvia falcata* Sp. Pl. Fl. Per. de Pillao”) was moved from the center of the specimen to the lower left-hand corner.

*Tofieldia frigida* was based on a collection or collections made by F.W.H.A. von Humboldt & A.J.A. Bonpland in Ecuador (“Crescit in frigidis regni Quitensis inter Loxam et pagum Ona, in summis montibus Saraguri, et Alpachacae, alt. 1200–1400 hexap, … Floret Decembri.”). While no material with this name was found in Paris (P-Bonpl.) (Stauffer et al. 2012), the type specimen of *Tofieldia flexuosa* in B-W is annotated as *Isidrogalvis* [sic] *frigida* Klotzsch, nom. nud. This strongly supports that the names *Tofieldia frigida* and *Tofieldia flexuosa* are based on the same gathering (i.e., *F.W.H.A. von Humboldt & A.J.A. Bonpland 3383*) and we have selected this collection to neotypify the name *Tofieldia frigida*.

### Harperocallis flava

McDaniel, J. Arnold Arbor. 49 (1): 38, figs 1, 2. 1968.

http://species-id.net/wiki/Harperocallis_flava

Fig. 2A, B

Isidrogalvia flava (McDaniel) Remizowa et al., Taxon 60 (4): 1092. 2011. Type: Based on *Harperocallis flava* McDaniel.

#### Type.

U.S.A. Florida: Franklin Co: 2.2 miles south of Sumatra, 11 May 1965, *S. McDaniel 6205* (holotype: A; isotypes: BM, DUKE, FLAS [FLAS-97596!], FSU [000004217!], GA, M!, MO!, NCU, NY!, PH [00013616!], SMU [BRIT 23682], US-not found).

#### Distribution

(Fig. 1). Endemic to the southeastern U.S.A. where it is known only from the Apalachicola River lowlands on the Coastal Plain of the Florida panhandle (Bay, Franklin, and Liberty counties).

#### Representative specimens.

**U.S.A. Florida:** Bay Co.: North of highway 22 and east of Star Avenue, 16 May 2003, *L. Keppner 37* (FSU [000081383]). Franklin Co.: 15 May 1978, *A.F. Clewell s.n*. (FSU [000004215]; 25 May 1979, *L.C. Anderson 4875* (FSU [000004220]). Liberty Co.: North side of turn in Route 65, 1.8 miles southwest of Wilma, 8 air miles north of Sumatra, 1 May 1986, *L.C. Anderson 9287* (FSU [000004223], NY); 12 May 1982, *A. Gholson, Jr. et al. 9699* (FLAS [FLAS-168245], FSU [000001249]); 19 Oct 2005, *L.C. Anderson et al. 23047* (FSU [000037492]). **District of Columbia:** U.S. Botanic Garden, Washington, cultivated from Florida, 7 Jul 2007, *L.M. Campbell 1045* (NY).

**Figure 2.**
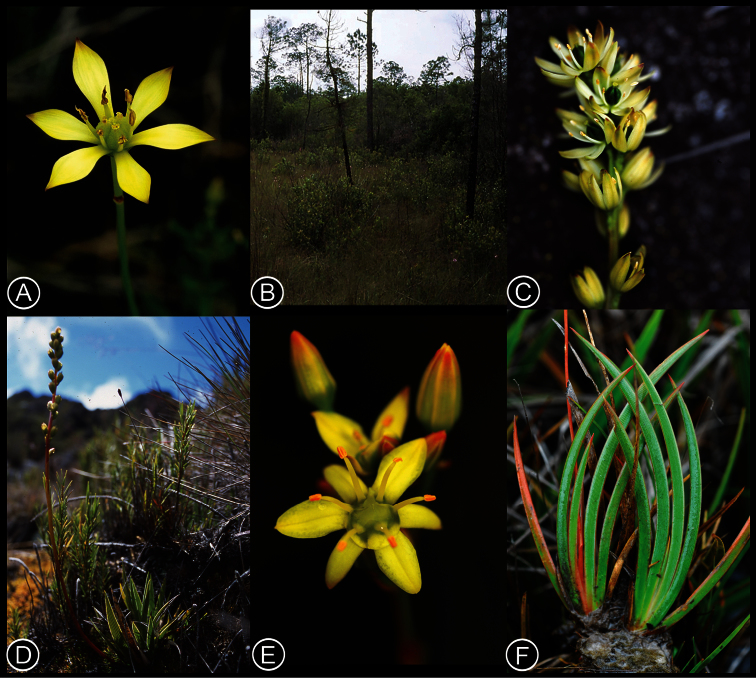
Habit and habitats of *Harperocallis*. *Harperocallis flava*. **A** Flower (note the peduncular bract, pronounced connective, and tuberculate ovary) **B** Habitat. *Harperocallis robustior*. **C** Inflorescence **D** Habit and habitat. *Harperocallis schomburgkiana*. **E** Flower **F** Leaves. (A, unvouchered, photograph A.R. Schotz; B, photograph G. Anglin, C, D *B. Stergios et al. 20368* (PORT), photograph K.J. Wurdack; E, F *K.J. Wurdack et al. 5636* (US), photograph K.J. Wurdack).

#### Note.

Zomlefer (1997) provides a complete description and detailed illustration of *Harperocallis flava*.

A search of the collection and accession records of the U.S. National Herbarium (US) indicates that not all of the herbaria McDaniel (1968) listed in his protologue have isotypes accessioned.

### Harperocallis longiflora

(Rusby) L.M. Campb. & Dorr comb. nov.

urn:lsid:ipni.org:names:77128360-1

http://species-id.net/wiki/Harperocallis_longiflora

Asagraea longiflora Rusby, Bull. New York Bot. Gard. 6 (22): 491. 1910. Type: Bolivia. La Paz: Franz Tamayo: Near Apolo, ca. 1785 m, 24 Jul 1902, *R.S. Williams 1471* (holotype: NY!).
Isidrogalvia longiflora (Rusby) Cruden & Dorr, Brittonia 44 (3): 368. 1992.

#### Type.

Based on *Asagraea longiflora* Rusby

#### Distribution

(Fig. 1). Known only from the type locality and vicinity in Bolivia (La Paz department); 1785–2000 m.

#### Representative specimen.

**Bolivia. La Paz:** Franz Tamayo: Senda Apolo–San José de Uchupiamonas, último arroyo antes de río Huacataya, 1958 m, 8 Oct 2002, *C. Maldonado et al. 3138* (MO).

#### Note.

Another recent collection, *C. Maldonado et al. 3121*, from the same locality at a slightly higher elevation (2000 m) is reported to be at LPB (see Missouri Botanical Garden 2013).

### Harperocallis neblinae

(Steyerm. ex L.M. Campb.) L.M. Campb. & Dorr comb. nov.

urn:lsid:ipni.org:names:77128371-1

http://species-id.net/wiki/Harperocallis_neblinae

Isidrogalvia neblinae Steyerm. ex L.M. Campb., Harvard Pap. Bot. 15 (1): 52, fig. 1. 2010. Type: Venezuela. Amazonas: Cerro de la Neblina, altiplanicie en la cumbre del brazo noroccidental, al norte del campamento base a lo largo del Río Mawarinuma, afluente del Río Baria, 1880 m, 7–8 Feb 1984, *J.A. Steyermark & J.L. Luteyn 129828* (holotype: VEN!; isotype: MO!).

#### Type.

Based on *Isidrogalvia neblinae* Steyerm. ex L.M. Campb.

#### Distribution

(Fig. 1). Known only from Cerro de la Neblina in the Guayana Highlands of Venezuela (Amazonas state); 1700–2100 m. This species is expected to occur also in the Brazilian part of the massif (Amazonas state).

#### Representative specimens.

**VENEZUELA. Amazonas**: Mpio. Río Negro, Cerro de la Neblina, altiplanicie en la cumbre del brazo noroccidental, al norte del campamento base a lo largo del Río Mawarinuma, afluente del Río Baria, aprox. 0°52–53'N; 66°05'W, 1880 m, 7–8 Feb 1984, *J.A. Steyermark & J.L. Luteyn* 129828-A (VEN-unicate); 4–6 km northeast of Cumbre Camp, 2100 m, 20 Nov 1957, *B. Maguire et al. 42154* (NY); Ridge at divide between Brazil and Venezuela, 26 km east-northeast of Neblina base camp, ca. 0°53'N, 65°56'W, 2000 m, 15 Apr 1984, *T. Plowman & W.W. Thomas 13594* (F).

### Harperocallis paniculata

(L.M. Campb.) L.M. Campb. & Dorr comb. nov.

urn:lsid:ipni.org:names:77128372-1

http://species-id.net/wiki/Harperocallis_paniculata

Isidrogalvia paniculata L.M. Campb., Harvard Pap. Bot. 15 (1): 52, fig. 2. 2010. Type: Brazil. Amazonas: Serra da Neblina, open slopes to base of cliffs, Pico Phelps, 2600–2700 m, 2 Dec 1965, *B. Maguire, J.M. Pires & C.K. Maguire 60463 p.p*. (holotype: NY!).

#### Type.

Based on *Isidrogalvia paniculata* L.M. Campb.

#### Distribution

(Fig. 1). Known only from Serra da Neblina in Brazil (Amazonas state); 2600–2700 m. This species may occur also in the Venezuelan part of the massif (Amazonas state).

### Harperocallis penduliflora

(L.M. Campb.) L.M. Campb. & Dorr comb. nov.

urn:lsid:ipni.org:names:77128373-1

http://species-id.net/wiki/Harperocallis_penduliflora

Isidrogalvia penduliflora L.M. Campb., Harvard Pap. Bot. 15 (1): 54, fig. 3. 2010. Type: Venezuela. Amazonas: Mpio. Río Negro, Cerro de la Neblina camp 2, Neblina massif, 2.8 km NE of Pico Phelps, 2100 m, 15 Apr 1984, *B.A. Stein & A.H. Gentry 1559* (holotype: VEN!; isotypes: K [K000400739!] US!).
Isidrogalvia schomburgkiana var. *patula* Remizowa, Byull. Moskovsk. Obshch. Isp. Prir., Otd. Biol. 112 (4): 74. 2007. Type: Venezuela. Amazonas: Mpio. Río Negro, Neblina Massif, Camp II, 2.8 km NE of Pico Phelps (= Neblina), 2085–2100 m, 16 Mar 1984, *B.L. Stannard 137* (holotype: K [K000400738!]; isotype: VEN!).

#### Type.

Based on *Isidrogalvia penduliflora* L.M. Campb.

#### Distribution

(Fig. 1). Known only from the vicinity of the type locality in the Guayana Highlands of Venezuela (Amazonas state); 1800–2100 m. This species likely occurs also in the Brazilian part of the massif (Amazonas state).

#### Representative specimens.

**VENEZUELA. Amazonas:** Mpio. Río Negro, Neblina massif, camp II, 2.8 km NE of Pico Phelps (= Neblina), on plateau, 0°49'40"N, 65°59'W, 2085–2100 m, 17 Mar 1984, *B.L. Stannard 166* (VEN); *S.S. Renner 2026* pro parte (MO [1434169]).

### Harperocallis robustior

(Steyerm.) L.M. Campb. & Dorr comb. nov.

urn:lsid:ipni.org:names:77128374-1

http://species-id.net/wiki/Harperocallis_robustior

Figs 2C, D , 3

Tofieldia sessiliflora var. *robustior* Steyerm., Fieldiana, Bot. 28 (1): 157. 1951. Type: Venezuela. Lara:Wet meadow at Las Sabanetas, above Los Aposentos, west of Humocaro Bajo, 2530 m, 5 Feb 1944, *J.A. Steyermark 55291* (holotype: F [F0046066F!]; isotype: NY!).
Isidrogalvia robustior (Steyerm.) Cruden, Syst. Bot. 16 (2): 278. 1991. Type: Based on *Tofieldia sessiliflora* var. *robustior* Steyerm.

#### Type.

Based on *Tofieldia sessiliflora* var. *robustior* Steyerm.

#### Distribution

(Fig. 1). Andes of Venezuela (Lara, Mérida, and Trujillo states); 2200–3700 m.

#### Representative specimens.

**Venezuela. Lara:** Mpio. Moran, below Páramo Las Rosas, 2285–3290 m, 25 Jun 1979, *R. Liesner et al. 7965* (MO, NY). **Mérida:** Mpio. Rangel, Cerro el Guamo, 3100–3200 m, 19 Jun 1988, *L.J. Dorr & L.C. Barnett 5627* (NY). **Trujillo:** Páramo de Cachaco, 3400–3700 m, 7 Nov 1927, *P. Christ 84* (NY); Mpio. Boconó, Páramo de Guaramacal, ca. 3080 m, ca. 9°14'N, 70°11'W, 28 Apr 1988, *L.J. Dorr et al. 4967* (NY, PORT); Páramo de Guirigay, 3000 m, 22 Apr 2002, *L.J. Dorr et al. 9200* (NY, PORT, US); Parque Nacional Guaramacal, vertiente norte, ca. 1977–2350 m, 28 Apr 1998, *B. Stergios et al. 17327* (PORT-unicate); Laguna Larga via Laguna Las Parias to Laguna Eco, Páramo de Motumbo, 2400–2600 m, 15 Sep 2003, *B. Stergios et al. 20368* (F, MO, NY, PORT, US).

**Figure 3.**
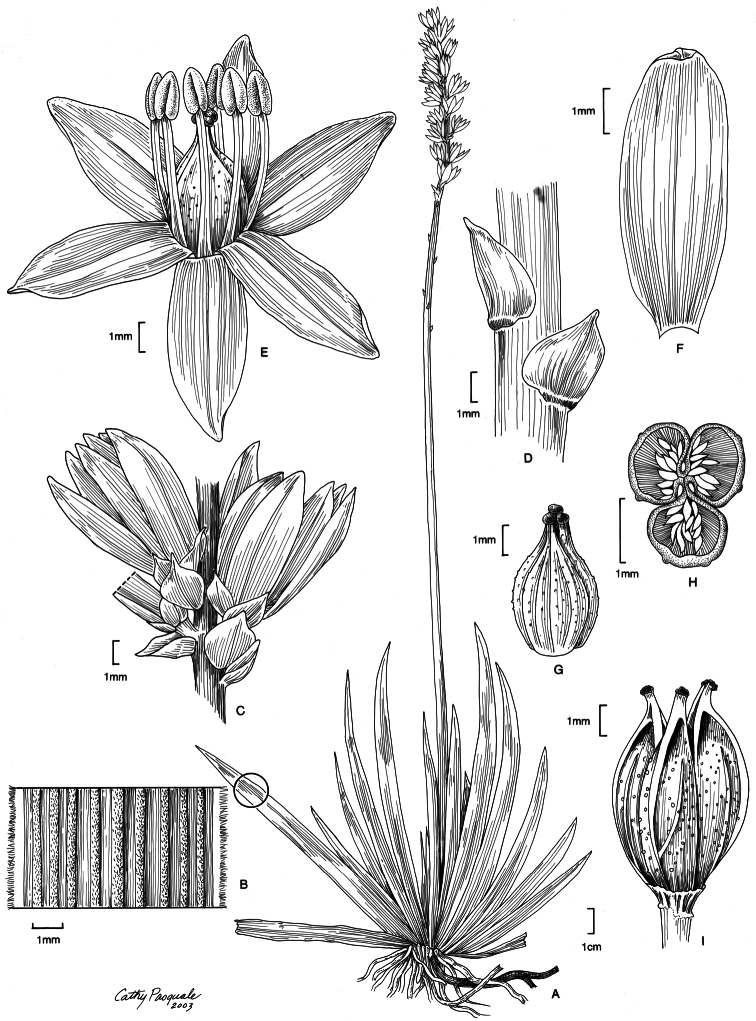
*Harperocallis robustior*. **A** Habit **B** Leaf (detail of parallel veins and tomentose margins) **C** Inflorescence (detail showing peduncular and calycular bracts subtending flowers) **D** Peduncular bracts (detail) **E** Flower **F** Tepal (adaxial view) **G** Ovary (note the tuberculae) **H** Ovary (cross-section showing axile placentation and ovules) **I** Capsule (carpels separating distally). (A, I from *L.J. Dorr et al. 4967* (US); B–H from *B. Stergios et al. 17327* (PORT).

### Harperocallis schomburgkiana

(Oliv.) L.M. Campb. & Dorr comb. nov.

urn:lsid:ipni.org:names:77128375-1

http://species-id.net/wiki/Harperocallis_schomburgkiana

Fig. 2E, F

Tofieldia schomburgkiana Oliv. in Thurn, Timehri 5: 206. 1886; [Trans. Linn. Soc., ser. 2, 2: 206, t. 49, fig. A 1–6. 1887]. Type: Venezuela. Roraima, summit, ca. 1845 m, Oct, *M.R. Schomburgk s.n*. (lectotype: K [K000099720!], MO [F negative 10002!], selected by Cruden 1991).
Isidrogalvia schomburgkiana (Oliv.) Cruden, Syst. Bot. 16 (2): 276. 1991. Type: Based on *Tofieldia schomburgkiana* Oliv.
Isidrogalvia guianensis Klotzsch in Ri. Schomburgk, Reis. Br.-Guiana 3: 1065. 1848 [1849], nom. nud.
Tofieldia guianensis (Klotzsch) R. Schulze, Bot. Jahrb. Syst. 17 (3–4): 330. 1893, comb. illeg.

#### Type.

Based on *Tofieldia schomburgkiana* Oliv.

#### Distribution

(Fig. 1). Guayana Highlands of Venezuela (Amazonas and Bolívar states) and adjacent Guyana; 1430–2800 m.

#### Representative specimens.

**GUYANA. Cuyuni-Mazaruni Region:** Mt. Maringma, summit, 2110 m, 15 Jun 2004, *H.D. Clarke et al*. *11717* (MO, NY, US); Paruima, 20 km W, Waukauyengtipu, 1430 m, 18 Jul 1997, *H.D. Clarke et al. 5855* (NY); Below 1st escarpment (of four) of Kamakusa Mt., 0–1 mi. SW of Ducking (1st) Camp [heading] to savanna, 5°45'58.9"N, 60°15'57.6"W, 637m, 15 May 2012, *K.J. Wurdack et al. 5636* (US). **Upper Takutu**- **Upper Essequibo:** Mount Roraima, summit, Autumn 1898, *J.J. Quelch & F. McConnell 657* (NY). **VENEZUELA. Amazonas:** Mpio. Atabapo, Cerro Marahuaca-Atuhua-Shiho, cumbre, parte aislada al Sur-Oeste del Cerro, 2450–2480 m, 9–10 Feb 1982, *J.A. Steyermark et al. 124367* (MO, NY), cumbre, sección noroccidental, 2500 m, 16 Feb 1981, *J.A. Steyermark et al. 124393* (MO, NY); Caño Sapo, summit of Mount Duida, 1920 m, Aug 1928 to Apr 1929, *J.A. Steyermark 690* (NY). **Bolívar:** Cerro Guaiquinima, Río Paragua, 1800 m, 29 Dec 1951, *J.J. Wurdack 32817* (MO, NY); North Valley, 1600–1700 m, 10–12 Jan 1952, *J.J. Wurdack 33039* (NY); macizo del Chimantá, sección nor-oriental del Acopan-tepui, 1950 m, 8–11 Feb 1985, *J.J. Pipoly et al. 7207* (NY).

### Harperocallis sessiliflora

(Hook.) L.M. Campb. & Dorr comb. nov.

urn:lsid:ipni.org:names:77128376-1

http://species-id.net/wiki/Harperocallis_sessiliflora

Tofieldia sessiliflora Hook., Icones Pl., ser. 2, 7: t. 691. 1844. Type: Colombia.“Andes of New Grenada,” 1842–3, *J.J. Linden 410* pro parte (lectotype: K, selected by Cruden, 1991; isolectotype: BM [BM000938091!]).
Asphodeleris sessiliflora (Hook.) Kuntze, Revis. Gen. Pl. 2: 706. 1891. Type: Based on *Tofieldia sessiliflora* Hook.
Isidrogalvia sessiliflora (Hook.) Cruden, Syst. Bot. 16 (2): 279. 1991. Type: Based on *Tofieldia sessiliflora* Hook.
Isidrogalvia moritziana Klotzsch ex Baker, J. Linn. Soc., Bot. 17 (103): 489. 1879, nom. nud., pro syn.
Tofieldia moritziana (Klotzsch ex Baker) R. Schultze, Bot. Jahrb. Syst. 17 (3–4): 330. 1893, comb. illeg.

#### Type.

Based on *Tofieldia sessiliflora* Hook.

#### Distribution

(Fig. 1). Andes of Venezuela (Mérida and Táchira states) and Colombia (Antioquia, Boyacá, César, Cundinamarca, Huila, Magdalena, and Norte de Santander departments); 2500–3200 m. Cruden (1991) also cites a dubious record (*J.A. Steyermark 54804*) from Ecuador (Loja), which we have not mapped.

#### Representative specimens.

**VENEZUELA. Mérida:** Páramo de los Conejos, 3300 m, 24 Jun 1953, *L. Bernardi 685* (NY); Mpio. Rivas Dávila, 25 km NE of La Grita, 2830 m, 15 Apr 1984, *J.L. Luteyn & M. Lebrón-Luteyn 9928* (NY). **Táchira:** NE side of Páramo de Táma, 2900–3200 m, 18 Oct 1978, *J.L. Luteyn et al. 5906* (MO, NY, US). **COLOMBIA. Boyacá:** Cordillera Oriental, Páramo de Belén, 3150 m, 6 May 1959, *H.G. Barclay & P. Juajibioy 7564* (MO, NY). **Cundinamarca:** Chapinero, near Bogotá, 3000–3100 m, 18–23 Sep 1917, *F.W. Pennell 2013* (NY, US). **Huila:** Río Balsillas, 2100–2200 m, 3–5 Aug 1917, *H.H. Rusby & F.W. Pennell 756* (MO, NY-2 sheets, US). **Magdalena:** Sierra de Perijá, east of Manaure, Sabana Rubia, 3000–3100 m, 6 Nov 1959, *J. Cuatrecasas & R. Romero Castaneda 25040* (US). **Norte de Santander/César**: Cerro de Oroque, 3700–3960 m, 19–21 May 1969, *H. García-Barriga & R.J. Mejía 19740* (NY, US).

### Harperocallis sipapoensis

(L.M. Campb.) L.M. Campb. & Dorr comb. nov.

urn:lsid:ipni.org:names:77128377-1

http://species-id.net/wiki/Harperocallis_sipapoensis

Isidrogalvia sipapoensis L.M. Campb., Harvard Pap. Bot. 15 (1): 57, figs 4, 5. 2010. Type: Venezuela. Amazonas: Mpio. Autana, Cerro Cuao–Sipapo, canyon on northeastern end of massif, ca. 1700 m, 14 Sep 2001, *L.M. Campbell, G.A. Romero-González & C. Gómez 798* (holotype: VEN!; isotypes: GH!, MO!, NY!, TFAV).

#### Type.

Based on *Isidrogalvia sipapoensis* L.M. Campb.

#### Distribution

(Fig. 1). Known only from Cerro Sipapo in the Guayana Highlands of Venezuela (Amazonas state); 1500–1700 m. This species likely occurs also on the geologically and floristically similar Cerro Autana, which is near the type locality.

#### Representative specimens.

**VENEZUELA. Amazonas:** Mpio. Atures, areniscas del Cerro Cuao, Caño Cabeza de Manteco, 73 km SE de Pto. Ayacucho, 5°06'N; 67°24'W, 1580 m, Sep 1989, *A. Fernández et al. 6207* (MO, NY, PORT); Serranía Sipapo, cumbre, 5°N; 67°30'O, 1500 m, 17 Feb 1981, *J.A. Steyermark et al. 124556* (VEN).

#### Excluded name.

*Isidrogalvia borealis* Ruiz & Pav. ex Steud., Nomencl. Bot. 839. 1821., nom. nud., pro syn. = ***Tofieldia palustris* Huds.**

## Supplementary Material

XML Treatment for Harperocallis

XML Treatment for Harperocallis duidae

XML Treatment for Harperocallis falcata

XML Treatment for Harperocallis flava

XML Treatment for Harperocallis longiflora

XML Treatment for Harperocallis neblinae

XML Treatment for Harperocallis paniculata

XML Treatment for Harperocallis penduliflora

XML Treatment for Harperocallis robustior

XML Treatment for Harperocallis schomburgkiana

XML Treatment for Harperocallis sessiliflora

XML Treatment for Harperocallis sipapoensis
